# Exploratory and territorial behavior in a reintroduced population of Iberian lynx

**DOI:** 10.1038/s41598-021-93673-z

**Published:** 2021-07-08

**Authors:** Carmen Rueda, José Jiménez, María Jesús Palacios, Antoni Margalida

**Affiliations:** 1 Fundación CBD-Habitat, FCBDH, 28002 Madrid, Spain; 2 Institute for Game and Wildlife Research, IREC (CSIC-UCLM-JCCM), 13005 Ciudad Real, Spain; 3 Junta de Extremadura, Dirección General de Sostenibilidad, Paseo de Roma s/n, Módulo C, 06800 Mérida, Spain

**Keywords:** Ecology, Conservation biology, Behavioural ecology, Biodiversity, Conservation biology

## Abstract

In reintroduction projects, an analysis of dispersal, exploratory movements and territorial behavior of the species concerned offers valuable information on the adaptive management of threatened species and provides a basis for the management of future reintroductions. This is the case of the Iberian lynx (*Lynx pardinus*) an endemic and endangered species reintroduced in Extremadura (Spain) in 2014. We analysed spatial data from 32 individuals just after their reintroduction. Our findings show exploratory movements sufficient to colonise and connect population nuclei within a radius of about 50 km of the reintroduction area. No significant differences were found in the exploratory movements capacity or in any directionality of males and females. Our results showed an effect of sex on the sizes of the territories established, as well as an inverse relationship between them and the time elapsed since release. No effects of rabbit abundance and lynx density on the size of territories are occurring during the early stages of reintroduction. On average, the territories of reintroduced individuals were less stable than those previously described in natural populations. Findings indicate that the reintroduced population has successfully been established but it takes more than 5 years to stabilize the territories in the area. Exploratory movements of reintroduced lynx can be large and in any direction, even when there is still a lot of high quality habitat available, which should be taken into account when reintroducing species, especially terrestrial carnivores.

## Introduction

Reintroduction programs to restore the populations of threatened species have become widespread in conservation projects, and are now a useful and effective tool to restore wild populations and improve species’ conservation status^1–5^. Reintroduction efforts may not be successful due to different factors related to habitat quality or reintroduction protocols^6,7^. Reintroduced populations usually arise from a small number of founders, so they are more susceptible to demographic stochasticity and reintroduction success can be compromised^8^. In small, reintroduced populations, space-use behavior may also determine which animals are able to reproduce, thus affecting the ultimate population size^9^ and reintroduction success^10^. In the medium and long terms, if these populations are not well monitored and managed, this can lead to increased inbreeding and reduced genetic diversity, introducing the possibility of a population extinction vortex^11^. The time invested and the economic costs of these projects can become limiting factors in guaranteeing the success of reintroduction and reinforcement programs^12^. Adaptive, evidence-based management is therefore required^13,14^ to optimise the human and economic resources invested and to increase the probability of a successful reintroduction^15^.

The endangered Iberian lynx (*Lynx pardinus*) is an endemic species in the Iberian Peninsula, and was widely distributed in the past^16^. During the twentieth century, its numbers declined greatly with many local extinctions and a significant reduction in its historical range^17,18^. The species was in a critical situation at the beginning of the twenty-first century, with a maximum estimated population size below 200 individuals^19^, distributed in two isolated populations. As a result, a set of conservation projects started, both in situ^20^ and ex situ^21^, with considerable economic investment and wide political and social support. Iberian lynx reintroductions have been carried out on the last years on Portugal, Extremadura, Andalusia and Castilla-La Mancha following the IUCN recommendations for reintroductions, with a high degree of success^22,23^. These initiatives provide the opportunity to study the reintroduced animals’ use of space, and exploratory and dispersal behavior in new environments with no previous settled population.

The long-range exploratory movements of reintroduced animals during the first stages are key to detect optimal areas, connected with the source area, where new nuclei can be created, as well as the main corridors to connect populations^24^. Iberian lynx dispersal parameters have been widely studied on the Doñana wild population^25,26^. Habitat selection as also been shown to be different among dispersal and territorial individuals^27–29^. During exploratory movements, lynx are able to use low quality habitats prioritizing finding a territory over territory quality. This implies that sometimes they are brought to cross sub-optimal habitats, not contemplated on the reintroduction design and with more risk of mortality, for the establishment of territory. Dispersal parameters may differ between populations^30^, and movement patterns of reintroduced individuals differ from those observed in natural populations^31,32^ which may have important management implications^33^.

Iberian lynx, and other lynx species, exhibit a land tenure territorial structure when the population reaches certain density and stability^34,35^. This structure may change if conditions vary due to natural processes or management actions^36^. Territory size of Iberian lynx on natural populations depends mainly on sex, having males larger territories than females, rabbit abundance, and habitat quality, with the smaller territories reported on areas with high rabbit abundance and shelter^34,37,38^. Many animal species, particularity mammalian carnivores, have good spatial memories and are able to use this ability to modify their use of space, becoming more efficient and increasing their fitness as they explore their surroundings^39,40^. Accordingly, territorial individuals would be expected to minimise their territory size during the first years following reintroduction, until the population reaches the stable territorial structure observed in natural populations.

Early-stage behavior of reintroduced lynx has been slightly studied on the pre-release enclosure^41^ and during the first year after release^42^, but long-term studies of reintroduced Iberian lynx populations are still scarce. During the first stages, the intensive monitoring of the population is crucial to take management decisions and guarantee reintroduction success^43^. In this paper, we analyse the movement data of 32 Iberian lynx during the first 5 years of their reintroduction into Extremadura, SW Spain (2014–2018). The main objectives were to characterise their long-range exploratory movements, the territory acquisition, and the stability of the settled territories as well as to analyse the territory size of the territorial individuals and the factors that determine it. The final objective was to provide tools for managers and conservationists to optimise the implementation of future reintroduction and reinforcement projects.

## Material and methods

### Study area

**Figure 1:**
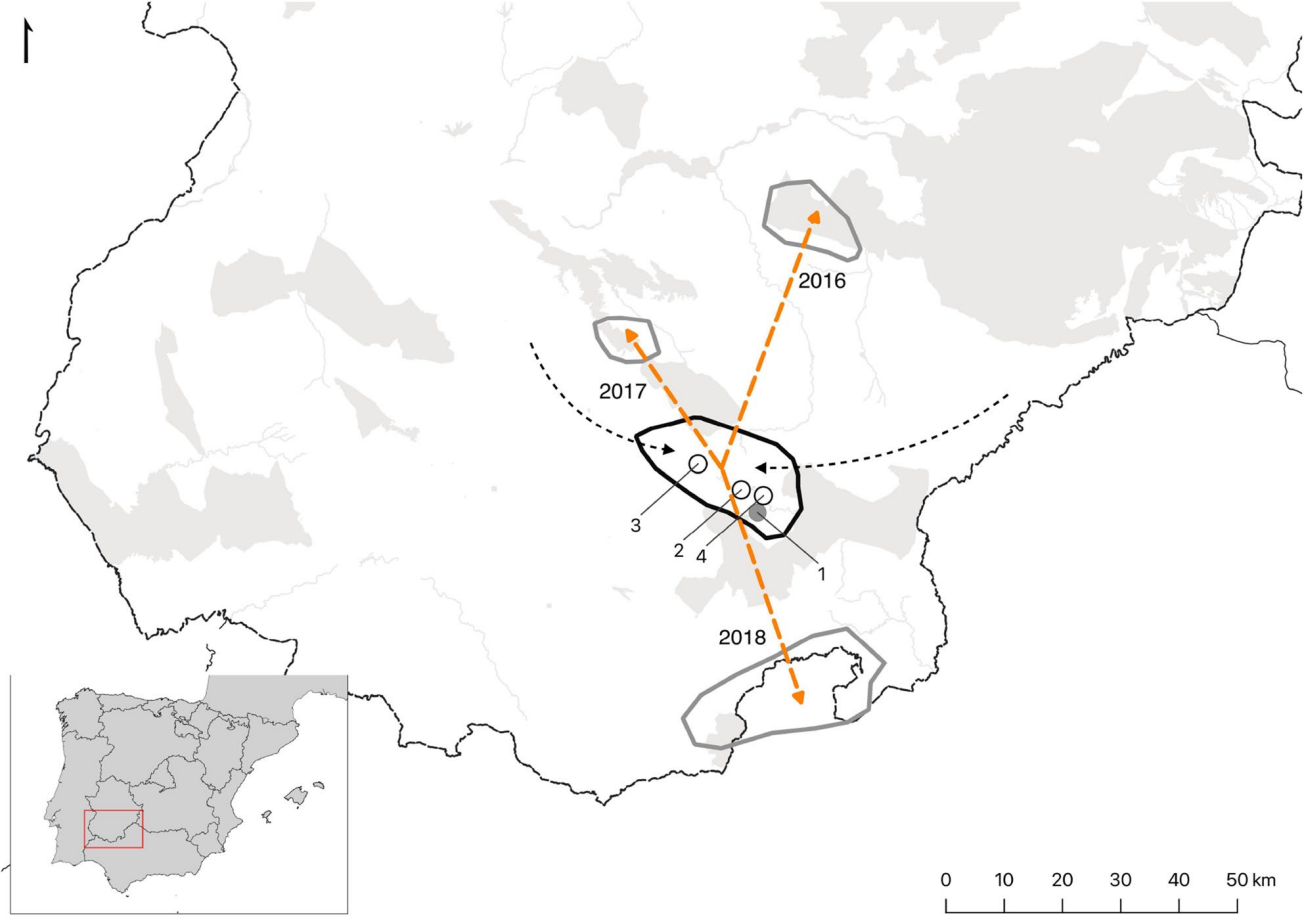
Study area showing the reintroduction area delimited by the black line, and colonized areas by grey lines. Release sites are represented (grey dot = soft release; white dot = hard release). Dashed line is the translocation of individual M03. The years in which stable territories were established in the colonized areas are indicated. Light grey polygons represent Nature 2000 Network. The maps were generated using QGIS 3.20.0 (https://www.qgis.org).

The study was carried out in the Iberian lynx reintroduction area of Hornachos-Matachel Valley in SW Spain (Badajoz, Extremadura) (Fig. 1) comprising some 90 km^2^. The Matachel Valley is centrally placed between the various lynx populations and could provide a strategically important area providing inter-connectivity between them^44^. The landscape mainly comprises Mediterranean woodland of evergreen oak (*Quercus ilex*), scrubland dominated by *Retama sphaerocarpa* and/or *Cistus ladanifer,* and small patches of open pasture and cropland*.* The climate is Mediterranean, characterized by very hot, dry summers. The area was selected for lynx reintroduction following the protocols of the LIFE + IBERLINCE project^45^ on the basis of its habitat quality (vegetation structure and rabbit abundance), social acceptability, and low risks of disease and non-natural mortality risks. The limits of the reintroduction area where based on continuously high rabbit abundance, which is the main limiting factor for lynx's territorial settlement. It should be noted that the habitats that surround the reintroduction area are completely different. Going north there is a continuous, more or less modified, Mediterranean woodland. To the South there is a large agricultural countryside comprising mainly cereal croplands, fallow lands, and pastures, with scant woody vegetation cover.

### Reintroduction and tracking

Between 2014–2018 thirty-two lynx were reintroduced on the area. Most of the reintroduced lynx (94%) were captive-bred juveniles around 1-year-old. In addition, a 2-year-old captive-bred male and a 9-year-old wild-born male, coming from the Doñana population, were released on 2014 and 2015 respectively. During the first 2 years of the reintroduction program, all lynx spent variable periods in a pre-release enclosure located within the reintroduction area (soft releases). Hard-releases, directly into the field, started in 2016 (Table 1) and were carried out on 3 different release points (Fig. 1), always selecting places with no settled territorial lynx.

**Table 1:** Reintroduced individuals in the Matachel Valley each year of the study period. Sex ratio M:F. Release method *S* soft, *H* hard. Days spent in the soft release enclosure (mean and range).

Year	N lynx	Sex ratio	Release method (S/H)	Days on the soft release enclosure
2014	8	5:3	8/0	10 (6–16)
2015	5	2:3	5/0	47 (21–127)
2016	9	4:5	3/6	12 (10–13)
2017	7	4:4	2/5	4 (2–6)
2018	3	1:2	0/3	–

**Figure 2:**
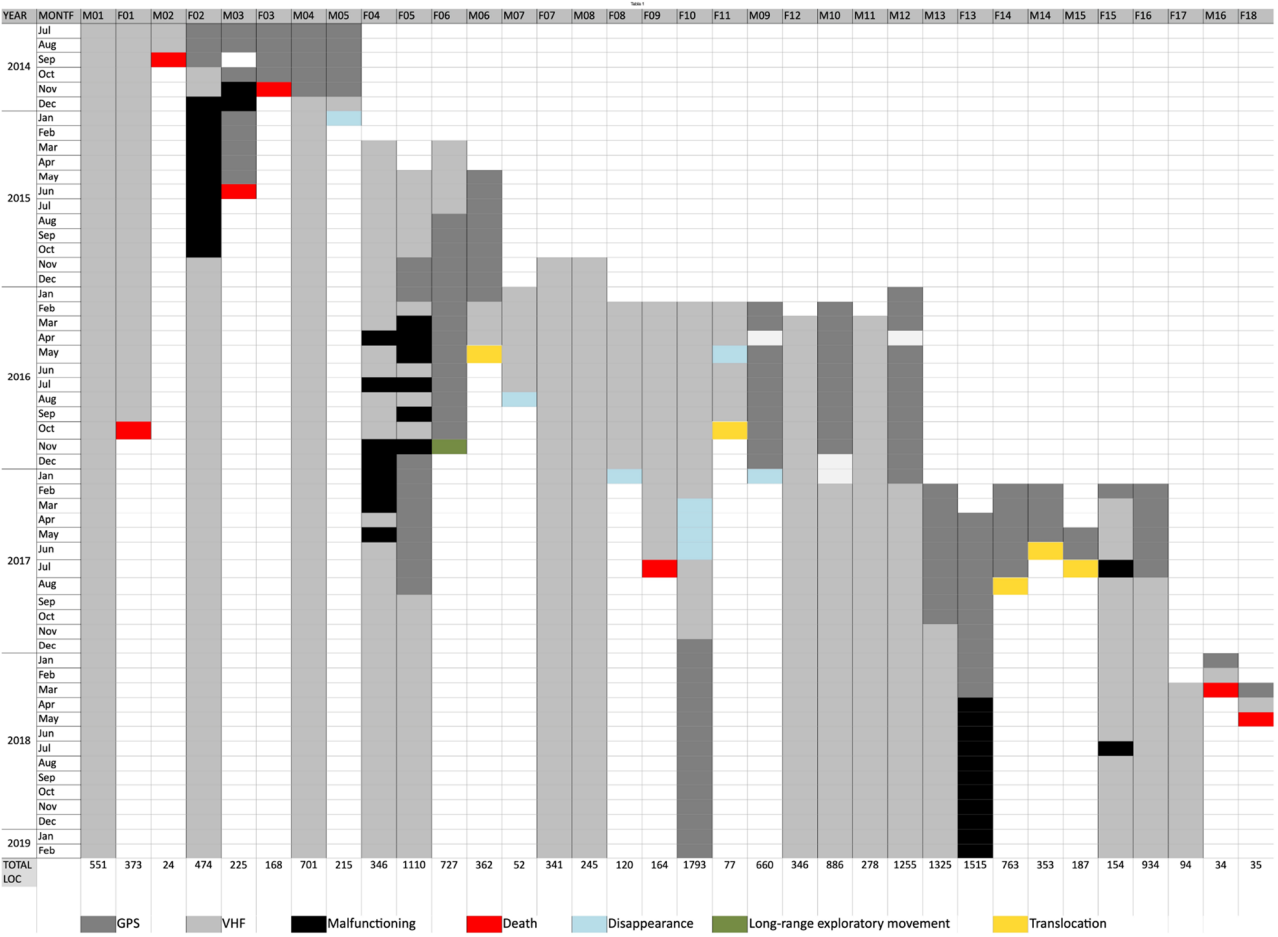
Tracking period of all the lynx included in the study. Collar type (GPS or VHF), and the cause of tracking interruption: collar malfunction, death, disappearance, or arrival to another subpopulation due to exploratory movements or translocation are also shown.

Reintroduced lynx were tagged with tracking collars during their last check up in the captive breeding centres before release. Lynx were captured to change malfunctioning collars and to tag wild-born animals using box traps, with an auxiliary wooden box. If possible, a warning device such as the *Minkpólice* or an e-mail notification camera trap (*Spartan*) was used. Capture and management protocols were applied following the Iberian lynx sanitary handbook developed by the Iberian Lynx Sanitary Aspects Advisory Panel^46^. Two methods were used to track the lynx (Fig. 2). GPS collars were used, when possible, during the first year following release, and were programmed to record 4–5 locations per day. Three different GPS-VHF collar models were used: Sirtrack G3C, Followit Tellus Ultra light, and PCB TM-202 L70. VHF collars were generally used after an individual had settled, and these lynx were located by triangulation^47^ 2–5 times per week. All the VHF collars used were Andreas Wagener Q-7.

### Long-range exploratory behaviour

To determine whether a lynx was resident or in an exploratory phase, a site fidelity analysis^48^ was performed, in four-monthly units. To evaluate the potential exploratory routes to connect the reintroduction area with other lynx populations, so long-range exploratory movements, we only considered exploratory movements outside the reintroduction area.

Twenty of the long-range exploratory events were tracked with GPS collars. In one of these cases (F15) the GPS transmitter failed eight days after exploratory movement started and it was tracked using VHF after this. Four individuals were tracked exclusively with VHF during their exploratory periods, with an average location frequency of 0.60 loc/day.

For each exploratory event, we calculated the duration of the exploratory period in days, the maximum distance (MD; distance between the last location on the reintroduction area and the farthest location recorded), the total distance covered (TD; the sum of the daily distances recorded between two consecutive locations), and the average daily distance covered (DD). The exploratory period was defined as the time elapsed between an animal starting an exploratory movement and then settling, temporarily or permanently, in a specific area, or it dying, or it being captured for translocation to a better-quality area.

Two cases of long-range exploratory movements were detected after the individuals settled on new distant areas, so no information was available on their actual exploratory period or route. In these two cases, the duration of the exploratory period, the total distance covered, and the average daily distance covered could not be calculated. Distance calculations were performed using ArcGIS 10.0. To determine if a movement from the reintroduction area had a specific directionality, we used the software Oriana v.4^49^ to calculate the main vector length (r) and the median angle (μ) of each event.

### Home range size and stability

An individual was considered resident if it showed site fidelity during at least a 4-month period in the reintroduction area, and territorial if site fidelity was maintained for at least 12 months. Individuals settled in territories outside the reintroduction area were excluded from the home range analysis, since they are isolated individuals, and these new areas have different characteristics in terms of available surface, habitat fragmentation and human disturbance. For the territorial behavior analysis, also two wild-born animals (F07 and M08), tagged on 2015, are included. Territorial stability was evaluated for each animal by comparing its home ranges in consecutive years (using minimum convex polygons excluding the most extreme 5% of the locations/MCP95) and applying the Cole index^50^ that estimates the degree of coincidence between any two areas.

We used two estimators to calculate home range size: the 95% minimum convex polygon (MCP95); and the 90% isopleth of a fixed kernel density estimator, using *h*_*ref*_ as bandwidth (K90). The core area was defined as the 50% isopleth of the kernel density estimator (K50). We considered two temporal scales. First, we calculated the seasonal home range (MCP95) for three 4-month periods (March–June; July–October; November–February) reflecting the different phenological stages of the species (breeding, post breeding and rut season)^34^. Second, we examined the annual home range and core area estimators (MCP95, K90, K50) for any evidence of the influences of sex, time since release, rabbit abundance, and lynx density on territory size.

### Prey abundance and resident lynx density

To estimate prey abundance, rabbit latrine counts^51^ were performed along fixed transects once a year, during May–September when rabbits were most abundant, to produce an annual abundance index (latrines/km) for each transect. The transects were distributed over a 1.25 km × 1.25 km grid, and generally ran along walkable paths through habitat suitable for rabbits. Each transect was at least 750 m long. The total number of transects sampled varied from 103–237 between years. To estimate the prey abundance in each lynx territory, we calculated the mean abundance index of all the tracks included in its home range^52^.

Due to the intensive monitoring of the lynx population using telemetry and camera trapping surveys, the total number of lynx and their home ranges in the reintroduction area was known with a high degree of certainty. To calculate annual lynx density in the reintroduction area we only considered those resident lynx, excluding < 1 year old lynx, that showed site fidelity for at least 4 months, taking the total area as the polygon including all the residents’ home ranges during that year.

### Statistical analyses

A site fidelity analysis was performed using the *rhr* package^53^ and home range size was calculated using the *adehabitatHR* package^54^ both in R^55^. Directional information on exploratory movements was used to perform a Rayleigh’s test of uniformity and a Rao’s spacing test using Oriana v.4. The Mann–Whitney–Wilcoxon test was used to determine whether there were significant differences between male and female home range sizes and exploratory activities. A Kruskal–Wallis test was performed to study the effect of the phenological period on territory size. We used general linear mixed models (GLMM) with a Gamma error distribution using the *lme4* package^56^ in R, with home range size (MCP95 and K90) and core area (K50) as the response variables. Animal identity was used as a random effect. Sex (S), rabbit abundance (RA), time since release (TsR), and lynx density in the area (LD) were included as covariates, as well as the interaction between sex and rabbit abundance. Candidate models were compared using the sample-size corrected Akaike information criterion (AICc) and AIC weights (*w*_*i*_) from the *MuMin* package^57^ in R. Values are presented as means with standard errors (SE).

### Ethics statement

All the work was conducted in accordance with relevant national and international guidelines, and conforms to all legal requirements in compliance with the Ethical Principles in Animal Research. Thus, protocols, amendments and other resources were conducted in accordance to the guidelines approved by the Extremadura Autonomous Government (Junta de Extremadura) following the R.D.1201/2005 (10 October 2005, BOE 21 October 2005) of the Ministry of Presidency of Spain. All experimental protocols were approved by the Extremadura Autonomous Government and MITECO (LIFE 10NAT/ES/570).

## Results

Between 2014–2018 a total of 16,887 locations were obtained for 34 different lynx. Eighteen of the 32 reintroduced lynx were tagged with GPS collars during the first months after release. GPS collars were also attached to three reintroduced individuals re-tagged during the exploratory period or when they were settled in remote areas on the very margins of the area covered by VHF telemetry. The average lifespan of the GPS collars was 180.4 days (SE: 24.67, range: 25–427 days). 27 lynx were located by means of VHF Telemetry, and this was the main method used to obtain fixes during the study period as a whole.

### Long-range exploratory behavior

**Figure 3:**
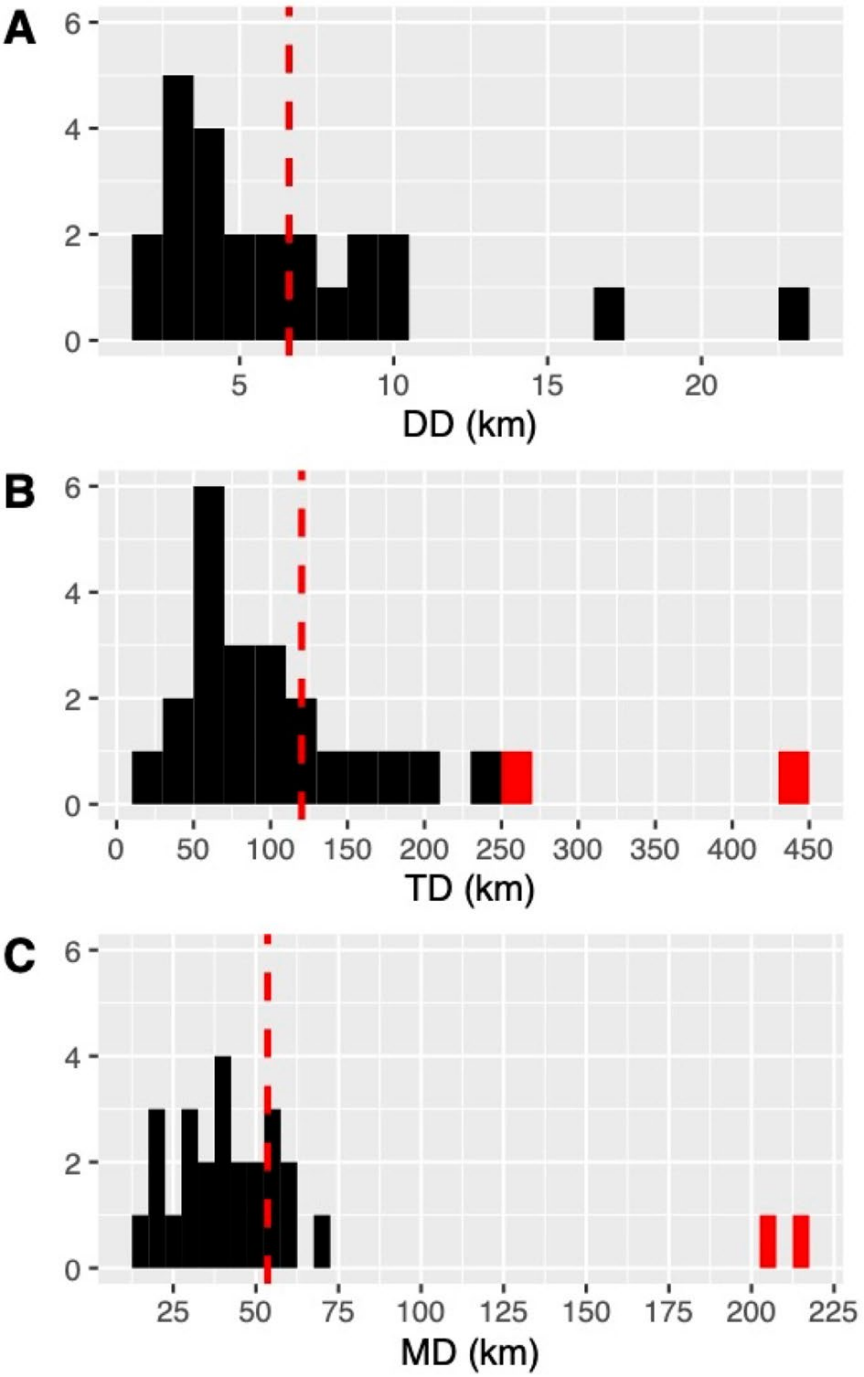
Long-range exploratory capacity results: (**A**) daily distance covered; (**B**) total distance covered; and (**C**) maximum distance covered. Means are represented by red dashed lines. Red bars represent individual F06.

We recorded 26 long-range exploratory events for 15 different individuals (8M:7F). Exploratory movements started on average 98 (SE: 22, range: 0–394) days after release. Most of the long-range exploratory events (96%; N = 25) occurred during the first year after release, with animals aged between 1 and 2 years old; and 65% (N = 17) started during the first 3 months after release. Long-range exploratory periods lasted an average of 29 days (SD: 5, range: 1–83). Most of the events (81%; N = 21) ended with temporary or final settlement of the individual in a specific area. The animal M03 was captured and translocated back to the reintroduction area in two occasions, one due to low body condition after crossing a sub-optimal area, and the other it was captured in a village on other sub-optimal area. In three cases, the animal died during the exploratory movement (M02 was poisoned and M03 and M16 were hit by vehicles). In two cases the animal was captured and translocated. The mean maximum distance (MD) was 53.54 km (SE: 9.53, range: 19.90–215.83 km) and the total distance covered (TD) was on average 120.21 km (SE: 18.65, range: 23.48–441.11 km). The mean daily distance travelled (DD) was 6.60 km (SE: 1.03, range: 1.50–23.48 km/day) (Fig. 3). There were no statistically significant differences between males and females for DD (Mann–Whitney–Wilcoxon test: W = 71, p = 1.0) or TD (Mann–Whitney–Wilcoxon test: W = 98, p = 0.134). In contrast, there were statistically significant differences between the MD of males and females: (female MD = 71.20 km, SE: 17.76); male MD = 35.88 km, SE: 3.27; Mann–Whitney–Wilcoxon test: W = 125, p = 0.039). Nevertheless, the farthest exploratory movements (MD = 216 and 207 km) correspond to the same female (F06), who was translocated back to the reintroduction area after its settlement on a sub-optimal and isolated area, and again performed a similar trajectory thereafter. If we exclude this female from the analyses, there were no statistically significant differences between the MD of males and females (Mann–Whitney–Wilcoxon test: W = 99, p = 0.12).

**Figure 4:**
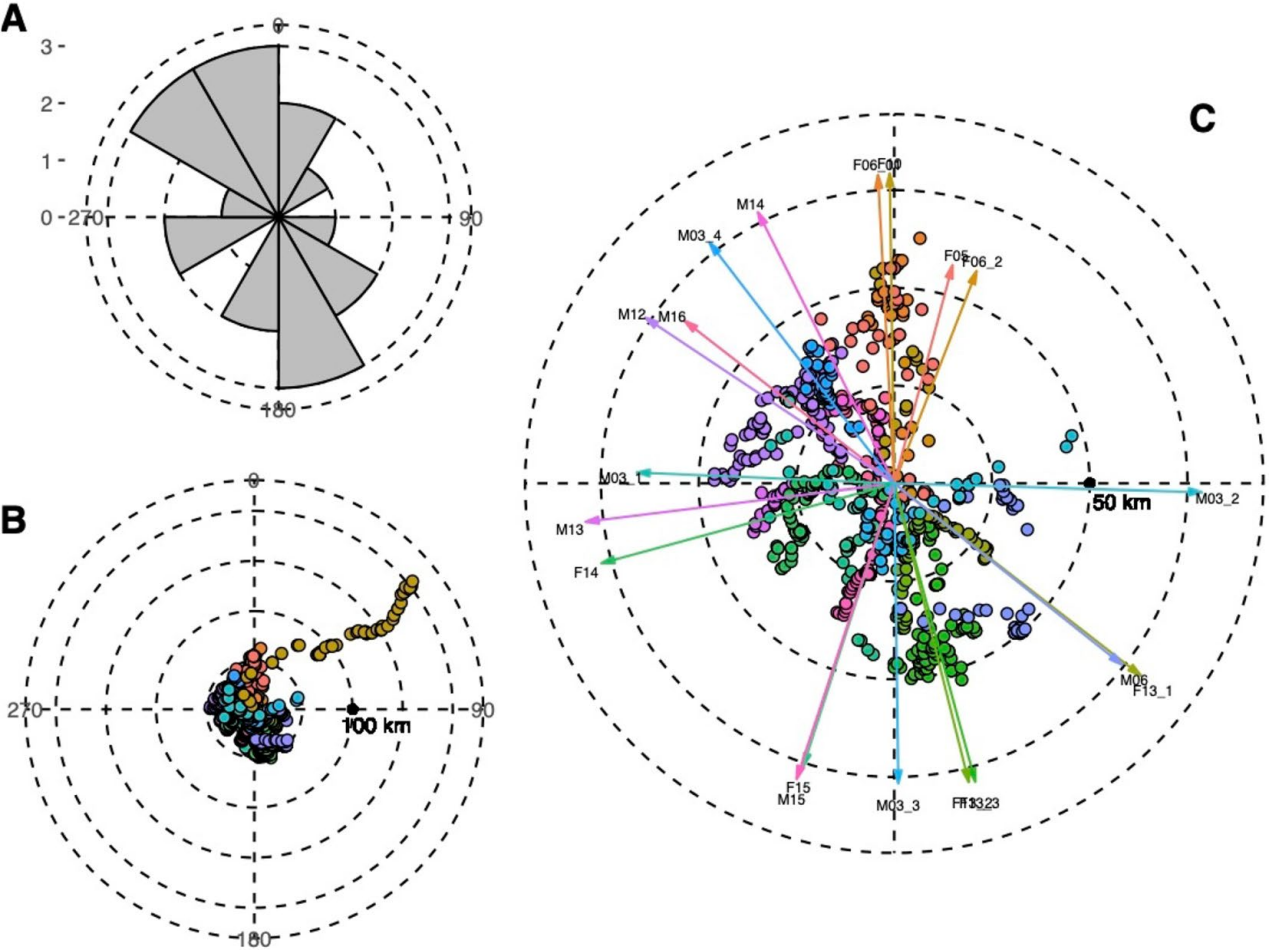
(**A**) Frequency distribution of mean angles (μ) of all long-range exploratory movements recorded from the reintroduction area. (**B**) Direction (in°) and distance from the centroid of the reintroduction area (in km) of all the locations of the exploratory movements. Each colour represents a different event. (**C**) Arrows represent the mean movement angle of each exploratory event (μ) and are proportional to the vector length (r). The longest exploratory event (F06) is omitted.

We recorded 20 long-range movements starting in the reintroduction area with enough registered locations to perform the directionality tests (Fig. 4b,c), corresponding to 13 different individuals (7M:6F). Each of the exploratory movements had a specific directionality (Rayleigh’s test p < 0.01 in all cases). The frequency distribution of the main vector’s mean angle of each movement produced a bimodal distribution N–NW (300°–30°) and S–SE (120°–210°), these being the principal exploratory directions in 75% of the cases (Fig. 4a). However, the hypothesis that exploratory movements overall are uniformly distributed cannot be rejected (Rayleigh test: Z = 0.264, p = 0.772, Rao’s spacing test: U = 131.353, p > 0.10).

### Territorial acquisition, stability and mortality

**Figure 5:**
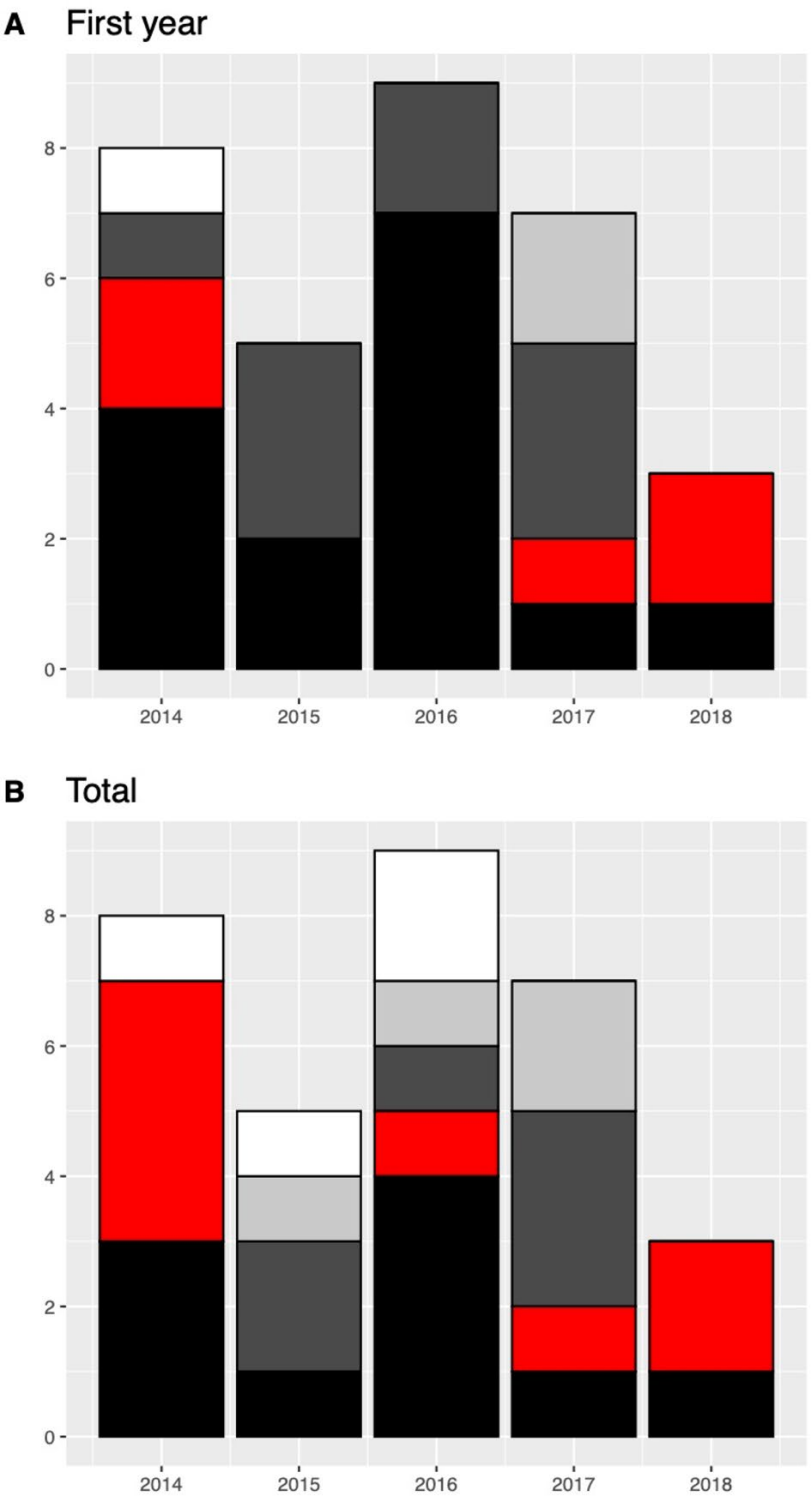
Status of the reintroduced lynx during the first year after release (**A**), and at the end of the study period (**B**). Five different statuses are shown: resident at the reintroduction area (black); found dead (red); dispersed to another area (dark grey) translocated to another area (light grey); and disappeared (white).

A total of 20 individuals (62% of the lynx released) established and maintained a territory to the end of 2018. Territorial individuals established their territories while 1–3 years old, and 94% of them started to show site fidelity to their future territories during the first year following release, aged between 1 and 2 years old. Thirty-four percent of the released lynx (N = 11) showed territorial behavior in the reintroduction area. At the end of the study period, 82% of them maintained the territory and 18% were found dead. Annual settlement success, calculated as individuals that acquired and maintained a territory in the reintroduction area/total number of individuals released, varied between years (2014 = 0.38; 2015 = 0.20; 2016 = 0.44; 2017 = 0.14; 2018 = 0; Fig. 5). Twelve percent of the animals released were able to find a territory in another area after a long-range exploratory movement (N = 4) and 16% obtained a territory on a recently colonized area following a translocation (N = 5).

Mean territory stability in the reintroduction area was 60% for females (range: 28–84%) and 53% for males (range: 20–86%). Mean stability did not vary between years ranging between 50–55%, except during 2015–2016 (75%).

During the study period at least eight wild-born lynx settled in territories in the reintroduction area. At the end of the study period, the territorial population comprised 17 individuals, 53% of which were captive-bred and 47% which were born in-situ.

Thirty-eight percent of the released individuals showed no territorial behavior: 55% of these died before establishing a territory; 36% disappeared; and 9% did not settle in a specific area for long enough to be considered as territorial. We recorded eight mortality events, representing 25% of the lynx released. Fifty percent of the mortality events occurred in the reintroduction area, and 50% of these occurred during an exploratory movement or after the lynx became established in another area. Causes of mortality were: being hit by a road vehicle (50%, N = 4); disease (25%, N = 2); poisoning (12.5%, N = 1); and unknown (12.5%, N = 1). Most of the deaths (75%) were recorded during the first year following release (mean: 135 days after release, SE: 44).

### Territory size

The home range size and core areas of males were significantly larger than female’s (Mann–Whitney–Wilcoxon, MCP95: W = 37, p = 0.002; K90: W = 50, p = 0.017; K50: W = 47, p = 0.011, Table 2). We could not find any significant differences in home range size according to phenological stage, for either males (Kruskal–Wallis: χ^2^ = 1.840, df = 2, p = 0.398) or females (Kruskal–Wallis: χ^2^ = 3.797, df = 2, p = 0.149).

**Table 2:** Iberian lynx territory size: the results of the analyses in this study and in other populations using VHF telemetry. *RB *Reserva Biológica subpopulation, *CR *Coto del Rey subpopulation. The values of home range are presented as mean (SE).

	Area	HR (km^2^) Males	HR (km^2^) Females	N lynx (M:F)	Period	HR estimator
This study	Matachel	5.62 (0.43)	3.40 (0.28)	6:7	Seasonal	MCP95
This study	Matachel	9.08 (0.81)	5.72 (0.57)	6:7	Annual	MCP95
This study	Matachel	13.17 (1.04)	9.83 (1.25)	6:7	Annual	K90
Ferreras et al. (1997)	Doñana (RB)	10.3 (1.9)	8.7 (2.4)	5:5	Seasonal	MCP95
Ferreras et al. (1997)	Doñana (RB)	16.9 (3.1)	12.6 (4.0)	4:5	Annual	MCP95
Palomares et al. (2001)	Doñana (CR)	10.3 (4.1)	5.3 (0.6)	3:4	–	MCP95
Gil-Sánchez et al. (2011)	Sierra Morena	12.0 (0.2)	5.9 (0.7)	4:6	Annual	MCP95
Sarmento et al. (2017)	Vale do Guadiana	11.18 (1.36)	9.87 (8.71)	4:4	Seasonal	K90

None of the best ranked models built to explain territory and core area size included either rabbit abundance or lynx density (Table 3). The measure of rabbit abundance in the territories varied from 32.32 latrines/km to 123 latrines/km (mean: 63.03, SE: 2.79). Variables with a statistically significant effect on territory size were: (1) sex, with a larger effect for males than for females, (MCP95, p = 0.002; K90 p = 0.024); and (2) TsR, the territories becoming smaller with increasing time after release (MCP95, p = 0.017; K90, p = 0.026). The variables affecting core area size were also sex, having the males bigger core areas than females (p = 0.040) and TsR, with the core areas becoming smaller with time (p = 0.039). The interaction between sex and rabbit abundance was only significant when using MCP95 as the territory size estimator, having the males larger territory size has rabbit abundance decreases (Table 4).

**Table 3:** Best ranked GLMM models relating territory size (MCP95 and K90) and core area (K50) with sex (S), time since release (TsR), rabbit abundance (RA), and lynx density (LD). Only models with ΔAIC_c_ < 2 are shown.

Model	K	AIC_c_	ΔAIC_c_	*w*_*i*_
**MCP95**				
S + TsR	5	136.33	0	0.68
TsR	4	137.81	1.48	0.32
**K90**				
S + TsR	5	167.39	0	0.46
TsR	4	167.84	0.45	0.37
S	4	169.34	1.95	0.17
**K50**				
S + TsR	5	100	0	0.29
TsR	4	100.22	0.22	0.26
Null	3	100.47	0.47	0.23
S	4	100.57	0.57	0.22

**Table 4:** GLMM models showing the effects of sex (S), time since release (TsR), rabbit abundance (RA), lynx density (LD) and the interaction between S and RA, on territory size (MCP95 and K90) and core area size (K50) using the global model. The effect of sex is given relative to females territory. Significance levels (**p < 0.01, *p < 0.05).

	Estimate (SE)		
	MCP95	K90	K50
Intercept	7.56 (1.81)**	12.91 (2.99)**	3.10 (0.95)**
TsR	− 1.10 (0.46)*	− 2.03 (0.91)*	− 0.55 (0.27)*
Sex(M)	5.57 (1.81)**	6.37 (2.82)*	1.88 (0.91)*
RA	2.26 (1.98)	1.06 (4.03)	1.13 (1.36)
LD	0.25 (1.27)	1.64 (2.33)	0.71 (0.65)
Sex(M):RA	− 6.24 (2.96)*	− 5.27 (5.29)	− 1.14 (1.82)

## Discussion

### Long-range exploratory behavior

In other wild felid reintroduction programs, reintroduced individuals usually make long-range movements and settle far away from the release site^24,58^. Soft releases and enough surface of available high-quality habitat may reduce initial exploratory behavior^59^. However, in our study, long-range exploratory movements occur from the first year of reintroduction, using soft-release enclosures, having high quality habitat available, and with low intraspecific competition, while other individuals settled in the reintroduction area just after their release. Other factors, such as place of release and personality of the individual, may be influencing the long-range exploratory behavior^6,60^. As expected, the average distance travelled by reintroduced lynx in our case study is greater than that recorded during natural dispersal in the Doñana wild population^25,26^.

Lynx coming from the release area settled into stable territories in three new colonized areas with high habitat quality and high rabbit densities, 30–40 km away in N, NW and S directions (Fig. 1). During 2015, all the colonized areas were visited by four different lynx for the first time. We recorded lynx reproduction in all these areas in 2019, indicating that subpopulations may be founding there, connected with the release site, and perhaps forming a single metapopulation in the future. Our results suggest that the exploratory behavior of lynx could be sufficient to maintain a regular flow of individuals between populations as much as 50 km away, or even farther, to mix with more distant populations. Although the exploratory routes appear to follow a bimodal distribution, the directionality analyses indicate that lynx exploratory directions are actually uniformly distributed. This suggest that reintroduced lynx are able to explore long distances in any direction, from early stages of the reintroduction, not following an habitat selection, and should be taken into account when designing and planning the monitoring of new reintroduction areas. Considering the exploratory patterns previously described for this species, the age of the reintroduced lynx, and the results of this study, future GPS monitoring of the reintroduced animals should focus on their first year following release, since most of the long-range exploratory movements and mortality events take place during this period. To evaluate subpopulation connectivity, it would be necessary to keep monitoring the population once it is finally stablished, with a particular focus on the natural dispersal of wild-born lynx.

### Territory acquisition and stability

During the first stages of a reintroduction the key factors governing successful establishment of a new population are settlement success, survival rate, and reproduction of territorial founders^61^. Survival rates of reintroduced Iberian lynx are higher than those recorded in another carnivore reintroductions^62^. In the initial stages of a reintroduction, there are few territorial individuals in the area to limit the settlement of sub-adult lynx, so the age of territorial acquisition is lower than in natural populations^34^. It seems there is a negative trend of settlement success over time, but fluctuations do occur. This might be due to stochastic processes, since the number of released individuals each year is low, the sample size is small and the observed trend should be interpreted with caution. Since wild-born lynx have a high tendency to philopatry, these individuals have high chances to fill optimal territories on the reintroduction area during the first years, hence limiting the settlement of subsequent reintroduced individuals. Although captive-bred Iberian lynx may have larger body sizes, especially females^63^, the wild-born animals have greater in-situ experience and knowledge of the area, which can provide a competitive advantage against reintroduced ones. In addition, some territorial species have a higher peripheral territory overlap with related conspecifics than they do with non-related individuals^64^. This may increase the probability that wild-born animals settle an adjacent territory. Considering that first founders occupy the best territories in terms of rabbit abundance^65^, this can also provide an advantage for wild-born lynx. Previous research on reintroduction biology indicates that wild-born animals may have higher survival rates and adaptive capacity than those born in captivity^31,62^. In other reintroduction projects, it has been shown that, after a certain time, released individuals do not contribute to the viability of the population^66–68^ and further releases after initial population establishment in an area is wasteful and strongly discouraged.

Regarding territory stability, in some systems territorial dynamics generate stable home ranges^37^. In our study, territorial stability is, on average, below the values described for natural populations^34^. It may well be that a 5-year period is not enough time for this reintroduction to reach the territorial stability of a mature population, especially given that new lynx were released each year which certainly had an effect on the territorial stability. Survival and reproduction of reintroduced lynx indicates a successfully established population, at least in the short term.

### Factors influencing territory size

The main factors influencing home range size in Iberian lynx are sex and age class, but also prey abundance^34,37^. Territory size of reintroduced lynx is slightly smaller, to the size recorded on other natural populations^34,37,69^ and similar to the one recorded on other reintroduced populations^42^. Our results show that sex and time since release (TsR) affect the territory size of reintroduced lynx. Since the two individuals released at more than 1 year of age did not establish territories, the TsR is always equivalent to the animal’s age in years. In any case, it is likely that the effect of TsR on territory size will only operate during the initial years of the reintroduction program, and will become less important as the territorial structure stabilizes. Reintroduced lynx select their territories based on those areas with the highest rabbit densities^65^, but once the lynx become territorial, our results suggest that rabbit abundance has no effect on the size of the territory. This is probably because the first territories are situated in areas where rabbit density is sufficient and exceeds the threshold above which increased food availability has no further effect on territory size. To study the effect of rabbit abundance on the size of lynx home ranges, it would be necessary to compare areas with greater and lesser ranges of rabbit densities. The differential response of males and females to rabbit abundance, although only significant regarding MCP95, may be due to the fact that females make more efficient use of space, looking for the best territories in terms of prey availability to raise their kittens, while males, having larger territories, probably due to patrolling and breeding behavior, thus including areas with less prey abundance.

Considering the effects of lynx density, it is possible that during the early years of the reintroduction, density did not influence the lynx’ use of space because the carrying capacity of the area had not yet been reached. The release of new individuals each year could have added some noise in the analyses as well. The average density of all resident lynx may not correctly represent the degree of competition for territories. It would be interesting to use the density of just territorial lynx, excluding resident lynx that showed site fidelity for less than 12 months, or the number of lynx of the same sex found in adjacent territories.

Our findings suggest that territories are not stable during the first years of the reintroduction, and they tend become smaller as time pass, allowing new territories to be stablished. Ideally these territories should be filled with selected reintroduced individuals, trying to achieve as much genetic variability as possible on the initial stages of the population founding.

It would be very interesting to evaluate the influence of individual lynx’s ‘personality’. Personality may exert a significant influence on processes such as dispersal or the use of home range areas^70^ as well as the survival of reintroduced individuals^71^. Additionally, pre-release, release and post-release management protocols may significantly influence survival, exploratory movement, and settlement rates^72,73^, although this is not always the case^74,75^. The effect of the release protocols, concerning type of release (soft vs. hard) and release point location, must be studied in depth.

Future studies of these issues are essential to improve our understanding of species reintroduction programs in particular, and conservation biology in general, and to develop better evidence-based reintroduction protocols. This knowledge will lead to more effective reintroduction programs, and optimise use of the available resources.

## Data Availability

The datasets used and analyzed during the current study are available from C.R. and M.J.P. on reasonable request.

## References

[1] Seddon PJ, Armstrong DP, Maloney RF (2007). Developing the science of reintroduction biology. Conserv. Biol..

[2] Seddon PJ, Griffiths C, Soorae P, Armstrong DP (2014). Reversing defaunation: Restoring species in a changing world. Science.

[3] Pérez I (2012). What is wrong with current translocations? A review and a decision-making proposal. Front. Ecol. Environ..

[4] Brichieri-Colombi TA, Moehrenschlager A (2016). Alignment of threat, effort, and perceived success in North American conservation translocations. Conserv. Biol..

[5] Swan KD, Lloyd NA, Moehrenschlager A (2018). Projecting further increases in conservation translocations: A Canadian case study. Biol. Conserv..

[6] Wolf C, Griffith B, Reed C, Temple S (1996). Avian and mammalian translocations: Update and reanalysis of 1987 survey data. Conserv. Biol.

[7] Breitenmoser U, Breitenmoser-Wursten C, Carbyn LN, Funk SM, Gittleman JL, Funk SM, Macdonald DW, Wayne RK (2001). Assessment of carnivore reintroductions. Carnivore Conservation.

[8] Vandel JM, Sthal P, Herrenschmidt V, Marboutin E (2006). Reintroduction of the lynx into the Vosges mountain massif: From animal survival and movements to population development. Biol. Conserv..

[9] Saltz D, Rowen M, Rubenstein DI (2000). The effect of space-use patterns of reintroduced Asian wild ass on effective population size. Conserv. Biol..

[10] Reynolds MH (2012). Space use and reintroduction of Laysan teal. Anim. Conserv..

[11] Gilpin ME, Soulé ME, Soulé ME (1986). Minimum viable populations: processes of species extinction. Conservation biology: The science of scarcity and diversity.

[12] Snyder NFR (1996). Limitations of captive breeding in endangered species recovery. Conserv. Biol.

[13] McCarthy MA, Armstrong DP, Runge MC, Ewen JG, Armstrong DP, Parker KA, Seddon PJ (2012). Adaptive management of reintroduction. Reintroduction Biology.

[14] Colomer MA, Oliva-Vidal P, Jiménez J, Martínez JM, Margalida A (2020). Prioritizing removal actions for the reintroduction of endangered species: Insights from bearded vulture simulation modeling. Anim. Conserv..

[15] Serrouya R (2019). Saving endangered species using adaptive management. Proc. Natl. Acad. Sci..

[16] Rodríguez A, Delibes M (1990). El lince ibérico (*Lynx**pardina*) en España: Distribución y problemas de conservación.

[17] Gil-Sánchez JM, McCain EB (2011). Former range and decline of the Iberian lynx (*Lynx pardinus*) reconstructed using verified records. J. Mamm..

[18] Casas-Marce M (2017). Spatiotemporal dynamics of genetic variation in the Iberian lynx long its path to extinction reconstructed with ancient DNA. Mol. Biol. Evol..

[19] Guzmán JN (2004). El lince ibérico (Lynx pardinus) en España y Portugal.

[20] Simón MA (2012). Reverse of the decline of the endangered Iberian Lynx: Saving the Iberian Lynx. Conserv. Biol..

[21] Vargas A (2008). The Iberian Lynx *Lynx pardinus* conservation breedingprogram: Iberian lynx conservation breeding program. Int. Zoo Yearbook.

[22] Simón MA (2017). Recuperación del lince ibérico en España y Portugal: situación actual de sus poblaciones.

[23] Aranda (2017). La reintroducción del lince ibérico en Castilla-La Mancha.

[24] Buderman F, Hooten MB, Ivan JS, Shenk TM (2017). Large-scale movement behavior in a reintroduced predator population. Ecography.

[25] Revilla E, Wiegand T, Palomares F, Ferreras P, Delibes M (2004). Effects of matrix heterogeneity on animal dispersal: From individual behavior to metapopulation-level parameters. Am. Nat..

[26] Ferreras P (2004). Proximate and ultimate causes of dispersal in the Iberian Lynx *Lynx pardinus*. Behav. Ecol..

[27] Palomares F (2000). Iberian Lynx in a fragmented landscape: Predispersal, dispersal, and postdispersal habitats. Conserv. Biol..

[28] Gastón A (2016). Response to agriculture by a woodland species depends on cover type and behavioural state: Insights from resident and dispersing Iberian Lynx. J. Appl. Ecol..

[29] Blázquez-Cabrera S (2016). Influence of separating home range and dispersal movements on characterizing corridors and effective distances. Landsc. Ecol..

[30] Ferreras P (2001). Landscape structure and asymmetrical inter-patch connectivity in a metapopulation of the endangered Iberian lynx. Biol. Conserv..

[31] Stoinski T, Beck B, Bloomsmith M, Maple TA (2003). Behavioral comparison of captive-born, reintroduced golden lion tamarins and their wild-born offspring. Behaviour.

[32] Margalida A (2013). Uneven large-scale movement patterns in wild and reintroduced pre-adult bearded vultures: Conservation implications. PLoS One.

[33] Berger-TAL O, Saltz D (2014). Using the movement patterns of reintroduced animals to improve reintroduction success. Curr. Zool..

[34] Ferreras P, Beltrán JF, Aldama JJ, Delibes M (1997). Spatial organization and land tenure system of the endangered Iberian lynx (*Lynx pardinus*). J. Zool..

[35] Benson JF, Chamberlain MJ, Leopold BD (2004). Land tenure and occupation of vacant home ranges by bobcats (*Lynx rufus*). J. Mamm..

[36] López-Parra M (2012). Change in demographic patterns of the Doñana Iberian lynx *Lynx pardinus*: Management implications and conservation perspectives. Oryx.

[37] Palomares F, Delibes M, Revilla E, Calzada J, Fedriani JM (2001). Spatial ecology of Iberian Lynx and abundance of European rabbits in Southwestern Spain. Wildl. Monogr..

[38] Simón MA (2012). Ten years conserving the Iberian lynx.

[39] Gautestad AO (2011). Memory matters: Influence from a cognitive map on animal space use. J. Theor. Biol..

[40] Powell RA, Boitani L, Powell RA (2012). Movements, home ranges, activity, and dispersal. Carnivore Ecology and Conservation.

[41] Figueiredo A (2019). Reintroduction of the Iberian lynx (*Lynx pardinus*): A preliminary case study in Extremadura, Spain. J. Ethol..

[42] Sarmento P, Carrapato C, Eira C, Silva JP (2017). Spatial organization and social relations in a reintroduced population of endangered Iberian Lynx *Lynx pardinus*. Oryx.

[43] Sarmento P, Carrapato C (2019). The use of spatially explicit capture-recapture models for estimating Iberian lynx abundance in a newly reintroduced population. Mamm. Biol..

[44] Blázquez-Cabrera S, Ciudad C, Gastón A, Simón MA, Saura S (2018). Identification of strategic corridors for restoring landscape connectivity: Application to the Iberian Lynx. Anim. Conserv..

[45] IBERLINCE Team Life+IBERLINCE Recovery of the historical distribution for Iberian Lynx (*Lynx pardinus*) in Spain and Portugal. (LIFE10NAT/ES/570) [WWW Document]. http://www.iberlince.eu/index.php/eng/project/description (2018).

[46] GAAS—Grupo Asesor de Aspectos Sanitarios del Lince Ibérico. Manual sanitario del lince ibérico. Versión 2.1. http://www.lynxexsitu.es/ficheros/documentos_pdf/85/Manual_Sanitario_Lince_Ib_2014.pdf (2014)

[47] White GC, Garrott RA (1990). Analysis of Wildlife Radio Tracking Data.

[48] Laver PN, Kelly MJ (2008). A critical review of home range studies. J. Wildl. Manag..

[49] Kovach WL (2011). Oriana-Circular Statistics for Windows, v.4.

[50] Cole LC (1949). The measurement of interspecific association. Ecology.

[51] Ferreira C, Paupério J, Alves PC (2010). The usefulness of field data and hunting statistics in the assessment of wild rabbit (*Oryctolagus cuniculus*) conservation status in Portugal. Wildl. Res..

[52] Monterroso P (2016). Disease-mediated bottom-up regulation: An emergent virus affects a keystone prey, and alters the dynamics of trophic webs. Sci. Rep..

[53] Signer J, Balkenhol N (2015). Reproducible home ranges (rhr): A new, user-friendly R package for analyses of wildlife telemetry data. Wildl. Soc. Bull..

[54] Calenge C (2006). The package “adehabitat” for the R software: A tool for the analysis of space and habitat use by animals. Ecol. Model..

[55] R Core Team. R: A language and environment for statistical computing. R Foundation for Statistical Computing. http://www.R-project.org/ (2020).

[56] Bates D, Mächler M, Bolker B, Walker S (2015). Fitting linear mixed-effects models using lme4. J. Stat. Soft..

[57] Barton, K. MuMIn—Multimodel Inference. R package version 1.43.6. http://CRAN.R-Project.org/package=MuMin (2019).

[58] Weise FJ (2015). Cheetahs (*Acinonyx jubatus*) running the gauntlet: An evaluation of translocations into free-range environments in Namibia. PeerJ.

[59] Briers-Louw WD, Verschueren S, Leslie AJ (2019). Big cats return to Majete Wildlife Reserve, Malawi: Evaluating reintroduction success. Afr. J. Wildl. Res..

[60] Yiu S (2015). Early post-release movement of reintroduced lions (*Panthera leo*) in Dinokeng Game Reserve, Gauteng, South Africa. Eur. J. Wildl. Res..

[61] Armstrong D, Seddon P (2008). Directions in reintroduction biology. Trends Ecol. Evol..

[62] Jule KR, Leaver LA, Lea SEG (2008). The effects of captive experience on reintroduction survival in carnivores: A review and analysis. Biol. Conserv..

[63] Reeves J, Smith C, Dierenfeld ES, Whitehouse-Tedd K (2020). Captivity-induced metabolic programming in an endangered felid: Implications for species conservation. Sci. Rep..

[64] Jackson CR, Groom RJ, Jordan NR, McNutt JW (2017). The effect of relatedness and pack size on territory overlap in African wild dogs. Mov. Ecol..

[65] Jiménez J (2019). Restoring apex predators can reduce mesopredator abundances. Biol. Conserv..

[66] Schaub M, Zink R, Beissmann H, Sarrazin F, Arlettaz R (2009). When to end releases in reintroduction programmes: Demographic rates and population viability analysis of Bearded Vultures in the Alps. J. Appl. Ecol..

[67] Bertolero A, Pretus JL, Oro D (2018). The importance of including survival release when assessing viability in reptile translocations. Biol. Conserv..

[68] Potts JR, Harris S, Giuggioli L (2012). Territorial dynamics and stable home range formation for central place foragers. PLoS One.

[69] Gil-Sánchez JM (2011). The use of camera trapping for estimating Iberian Lynx (*Lynx pardinus*) home ranges. Eur. J. Wildl. Res..

[70] Spiegel O, Leu ST, Bull CM, Sih A (2017). What’s your move? Movement as a link between personality and spatial dynamics in animal populations. Ecol. Lett..

[71] Bremner-Harrison S, Prodohl PA, Elwood RW (2004). Behavioural trait assessment as a release criterion: Boldness predicts early death in a reintroduction programme of captive bred swift fox (*Vulpes velox*). Anim. Conserv..

[72] Devineau O, Shenk TM, Doherty PF, White GC, Kahn RH (2011). Assessing release protocols for Canada Lynx reintroduction in Colorado. J. Wildl. Manag..

[73] Reading RP, Miller B, Shepherdson D (2013). The value of enrichment to reintroduction success. Zoo Biol..

[74] Hardman B, Moro D (2006). Optimising reintroduction success by delayed dispersal: Is the release protocol important for hare-wallabies?. Biol. Conserv..

[75] Sarkar M (2016). Movement and home range characteristics of reintroduced tiger (*Panthera tigris*) population in Panna Tiger Reserve, central India. Eur. J. Wildl. Res..

